# To Tell or Not: The Chinese Doctors’ Dilemma on Disclosure of a Cancer Diagnosis to the Patient

**Published:** 2018-11

**Authors:** Hongchun WANG, Fang ZHAO, Xiangling WANG, Xiaoyang CHEN

**Affiliations:** 1. Qilu Hospital, Shandong University, 107 Wenhua Xi Road, Jinan 250012, China; 2. Mayo Clinic, Minnesota, USA; 3. UT Southwestern Medical Center, TX, USA; 4. Humanistic Medicine Research Center, Shandong University, 107 Wenhua Xi Road, Jinan 250012, China

## Dear Editor-in-Chief

How to tell cancer patients about their diagnosis is approached in different ways across various countries and cultures. For example, in European and North American societies, physicians prefer direct truth telling (1), while in Asian countries, indirect truth telling is more culturally accepted (2). Families usually have major influence over clinicians’ disclosure of diagnosis to patients (3).

To investigate the opinions of clinicians in China regarding the disclosure of a cancer diagnosis and the underlying reasons for those opinions, a survey was administered to 180 physicians in Shandong Province in China to obtain the attitudes and relevant reasons regarding the disclosure of a cancer diagnosis to a patient.

We found that it remains difficult for clinicians in China to disclose diagnosis of a cancer, although autonomy has been recognized as an important ethical principle in medical practice. Our study shows that the diagnosis of cancers is often concealed from the patients, but disclosed to the families in China, for most physicians choose to inform the family first, but not the patients, generally based on the intention and desire of the family members, who prefer to conceal the diagnosis to the patients, considering the diagnosis would be a hard blow to the patients psychologically.

This study shows majority (98%) of the physicians would discuss the cancer diagnosis with family members before discussing it with the patient, and 82% of them will not tell the patient if the family requests “not to tell”. This phenomenon has been deeply rooted in the traditional Chinese culture -- Confucianism.

Confucianism regards human relations, rather than individual rights, as the basis of morality. The notion of the family (Jiā in Chinese) has a central position in Confucian classics, and the family is the basic autonomous unit. According to the Confucianism, when a patient was diagnosed with a cancer, his/her family members would deem it as their responsibility and even their natural right to make medical decision. If they think that it is harmful to inform the diagnosis to the patient, they usually request the doctor to dilute the severity of the disease, delay to tell, and even completely hide the diagnosis from the patient (4).

Our data shows that if the patients happened to be members of the physicians’ own families, nearly half of physicians would want to withhold the diagnosis. Modern Asian societies including China has been experiencing a transition, mainly due to the influence of the Western culture. Contemporary Chinese culture is a mixture of many values and beliefs from the ancient to the modern, and from the Western to the Eastern. Since the second half of the 1990s, more Japanese patients have received disclosure of a diagnosis of cancer than before (5). Most of the patients in China today hope their doctor to tell them the truth even if they have been diagnosed with cancer. Our study shows that if physicians themselves were diagnosed with cancer, 81% of them would want to know the diagnosis. There have been arguments whether the “Chinese family-based model” should be replaced by the Western model in order to respect the “autonomy” of Chinese patients. On the other hand, some argue that Asian bioethicists must develop a bioethics responding to their own cultural context (6).

In light of the current tendency towards more disclosure globally, and the fact that the majority of cancer patients in China want to know their diagnosis, the authors proposed a suggestion for resolution based on traditional “Chinese family-based model”. Before the diagnosis is disclosed, the doctor, the patient, and the family should discuss about who will be informed first, regardless of whether the diagnosis is good or not. Indeed, it is the patient’s right to decide whether to know or not.

So the decision should be highly rely on the intention of the patient himself/herself. If the patient choose “to be informed”, he or she should first discuss with their family members, which might be helpful to make a better decision. They can also ask advice from the doctor, who can help them to make a more reasonable decision from more professional point of view.
